# Calyculin A, an enhancer of myosin, speeds up anaphase chromosome movement

**DOI:** 10.1186/1475-9268-6-1

**Published:** 2007-03-24

**Authors:** Lacramioara Fabian, Joanna Troscianczuk, Arthur Forer

**Affiliations:** 1 Department of Biology, York University, Toronto, Ontario, M3J 1P3, Canada

## Abstract

Actin and myosin *inhibitors *often blocked anaphase movements in insect spermatocytes in previous experiments. Here we treat cells with an *enhancer *of myosin, Calyculin A, which inhibits myosin-light-chain phosphatase from dephosphorylating myosin; myosin thus is hyperactivated. Calyculin A causes anaphase crane-fly spermatocyte chromosomes to accelerate poleward; after they reach the poles they often move back toward the equator. When added during metaphase, chromosomes at anaphase move faster than normal. Calyculin A causes prometaphase chromosomes to move rapidly up and back along the spindle axis, and to rotate. Immunofluorescence staining with an antibody against phosphorylated myosin regulatory light chain (p-squash) indicated increased phosphorylation of cleavage furrow myosin compared to control cells, indicating that calyculin A indeed increased myosin phosphorylation. To test whether the Calyculin A effects are due to myosin phosphatase or to type 2 phosphatases, we treated cells with okadaic acid, which inhibits protein phosphatase 2A at concentrations similar to Calyculin A but requires much higher concentrations to inhibit myosin phosphatase. Okadaic acid had no effect on chromosome movement. Backward movements did not require myosin or actin since they were not affected by 2,3-butanedione monoxime or LatruculinB. Calyculin A affects the distribution and organization of spindle microtubules, spindle actin, cortical actin and putative spindle matrix proteins skeletor and titin, as visualized using immunofluorescence. We discuss how accelerated and backwards movements might arise.

## Background

Mechanisms of chromosome movements during anaphase have been investigated extensively and several models attempt to explain the forces involved [1-4]. Proteins implicated as key players in mitosis include *tubulin *[5-7], *microtubule motors *[8-12], *actin *[1,13-16], *myosin *[1,15-22], the elastic component *titin *[23-25], and *matrix proteins *skeletor [16,22,26-28], megator [29], chromator [30], EAST [31,32], NuMA [33-37] and laminB [38]. In this article we present data dealing with spindle myosin.

Myosin in mitotic cells generally is thought to be involved with cytokinesis, primarily with contractile ring formation and ingression [39,40], and with positioning and orientation of the mitotic spindle [41]. But myosin also is present in the spindle [1,15]. Some of the early studies that showed that actin and myosin were present in the spindle also discussed a possible role for myosin in force production during anaphase chromosome movement [17,18,42-45], but no physiological data were presented. More recent evidences that implicate myosin function in anaphase chromosome movements are based on experiments using various inhibitors of myosin or inhibitors of myosin phosphorylation [1,21,22,46]. In particular, movement of chromosomes during anaphase is stopped or slowed by the myosin inhibitor 2,3-butanedione monoxime (BDM) [1,16,21] or by the Rho-kinase inhibitor Y27632 [1]. Our present experiments utilise Calyculin A (CalA), a compound which prevents myosin dephosphorylation.

In order for non-muscle and smooth muscle myosin to be functional, the regulatory light chain (RLC) of myosin must be activated by phosphorylation by specific kinases, either myosin light chain kinase (MLCK) [47-49] or Rho-kinase (Rho-K) [40,50-52], and possibly others [e.g. [53-55]]. Myosin homeostasis is achieved by the balance between activation by phosphorylation, and inactivation by dephosphorylation, the latter being due to the action of myosin light chain phosphatase (MLCPase) [40,56-58], a type 1 protein phosphatase (PPase1) [59], which, like most PPases1, is probably targeted to its site by activity of other proteins [60,61]. Rho-K plays a double role in myosin homeostasis: it phosphorylates myosin RLC, thereby MLRC is activated [62-66], and it phosphorylates MLCPase, thereby MLCPase is inactivated. Rho-K thus regulates the degree of myosin phosphorylation and hence the activity of myosin [52,67-69].

**Figure 1:**
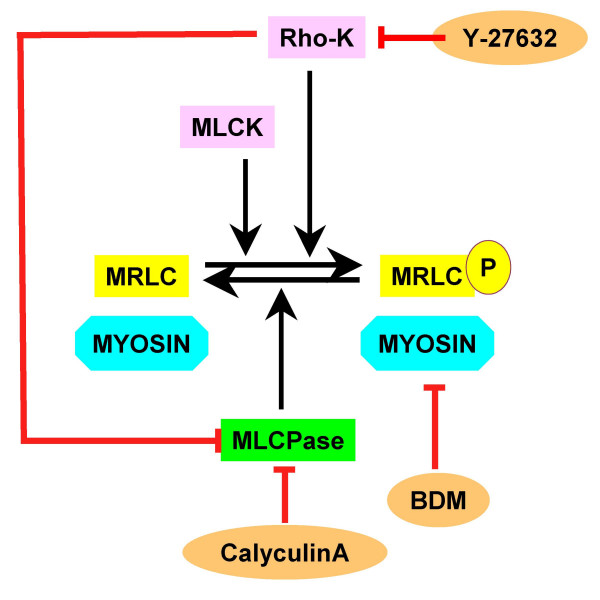
Schematic diagram showing the relationships between myosin and its activators and inhibitors. In order for myosin to be active, its regulatory light chain (MRLC) has to be phosphorylated either by myosin light chain kinase (MLCK) or by Rho-associated kinase (Rho-K). Dephosphorylation of MRLC is done by myosin light chain phosphatase (MLCPase). Various inhibitors interfere with myosin activity: Rho-K is inhibited by Y-27632, MLCPase is inhibited by Calyculin A and myosin is inhibited by BDM.

MLCPase is blocked by CalA, an inhibitor of serine/threonine phosphatase 1 and 2A [70-72] isolated from the marine sponge *Discodermia calyx *[73]. When MLCPase is inhibited myosin remains activated (Fig. 1), with an increased level of phosphorylation [74,75]. Thus, CalA activates actomyosin [76,77] and stimulates muscle contraction [68,70]. CalA has a variety of effects when applied to non-muscle cells, most of which are directly attributable to effects on myosin. For example, it causes contraction of stress fibres and cell cortex [72], stimulates retrograde flow and increases convergence of F-actin [78,79], induces actin and myosin aggregates [80-83], induces cleavage-like activity in cell cortices [76,84], and interferes with normal progression of the cleavage furrow [85]. We decided to study the effects of CalA on chromosome movement because previous studies that implicated myosin in anaphase force production were based only on results using inhibitors of myosin [16,21]. We reasoned, therefore, that a compound such as CalA that permanently activates (or hyperactivates) myosin might increase the poleward velocity of anaphase chromosomes. As we report in this article, CalA accelerates anaphase chromosome movements during anaphase and also has some unpredicted effects. Okadaic acid at the same concentration as CalA had no effect on chromosome movements. Since okadaic acid inhibits type 2 protein phosphatase (PP2A) at the same concentration as CalA [70], chromosome acceleration is not due to general effects on phosphatases such as PP2A. We interpet acceleration as due to hyper-phosphorylation of myosin.

## Results

### Control spermatocytes

**Figure 2:**
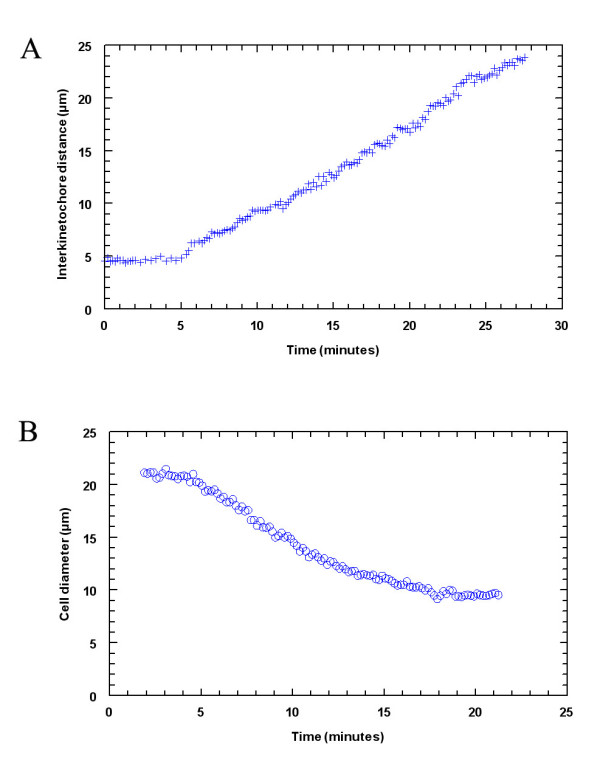
Plots of chromosome separation versus time during anaphase (A) and cell diameter at the site of cleavage furrow during cytokinesis (B) in a control crane-fly spermatocyte. One half-bivalent pair is shown in (A), illustrating constant separation velocity during anaphase.

In control crane-fly spermatocytes autosomal chromosomes move poleward in anaphase with constant separation velocities that average 1.2 μm/min (Fig 2A) [see Additional file 1]. After the autosomes reach the poles, the two equatorial univalent sex chromosomes start their own anaphase, moving poleward with much slower speeds of about 0.3 μm/min [86]. During sex chromosome anaphase the spindle also elongates [87]. Cytokinesis generally starts before sex-chromosome anaphase is completed and lasts about 10–20 minutes (Fig. 2B) [88].

### Calyculin A added in anaphase

We tested various concentrations of CalA, from 5 nM to 0.5 μM, in order to determine the lowest concentration that affects chromosome movement. Concentrations of 5 nM and 10 nM had no effect on anaphase chromosome movement, 20 nM had inconsistent effects, and 50 nM had consistent effects. Thus we used 50 nM for most of the experiments; unless noted otherwise, all descriptions are for cells treated with ≥ 50 nM CalA.

**Figure 3:**
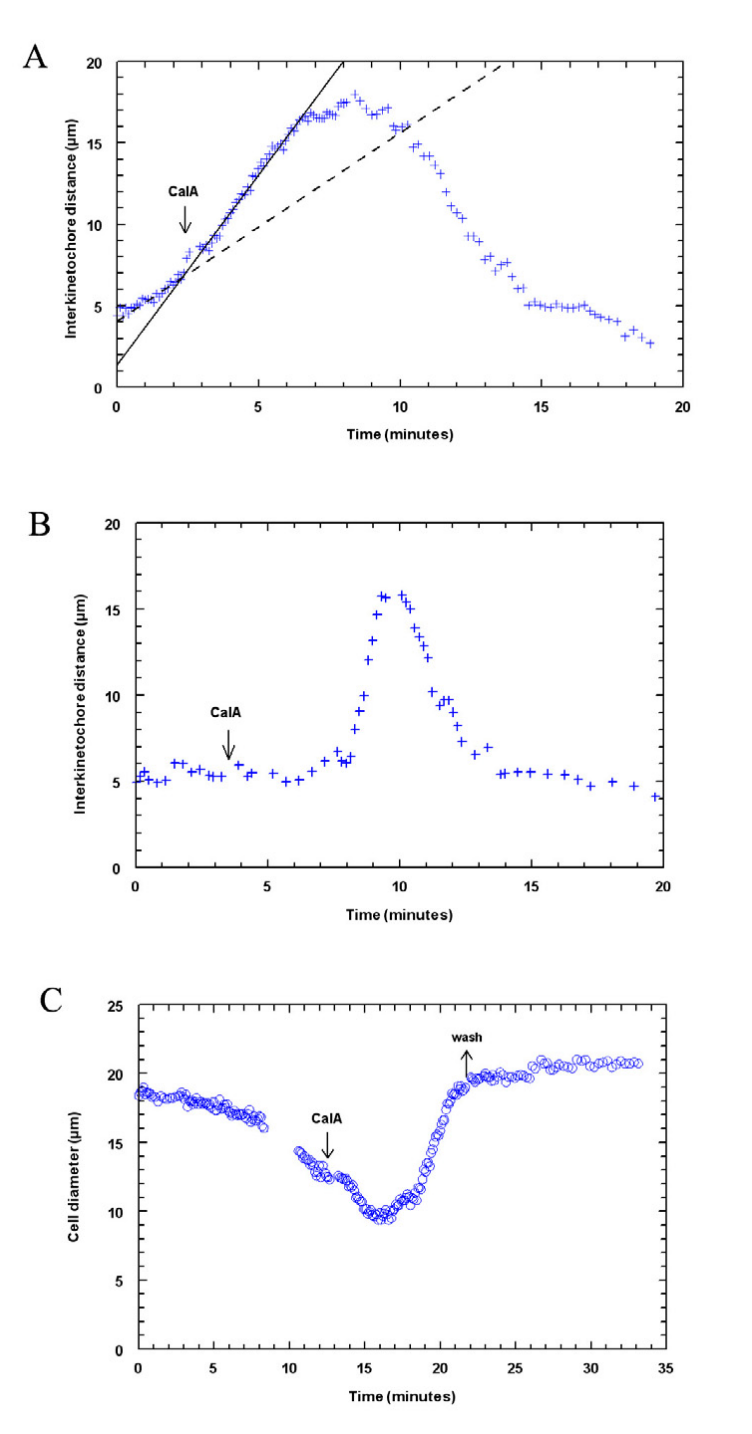
Calyculin A effects on anaphase chromosome movement and cytokinesis in crane-fly spermatocytes. **(A) **CalA added during anaphase (arrow) causes chromosomes to accelerate and to move backwards after they reached the poles (one half-bivalent pair is shown). Dashed line represents linear regression through the crosses before addition of CalA and the solid line after addition of CalA. **(B) **CalA added during metaphase (arrow), a few minutes before anaphase onset, causes chromosomes to move fast during subsequent anaphase and to move backwards after reaching the poles (one half-bivalent pair is shown). **(C) **CalA added during cytokinesis (downward arrow) stops furrow ingression and reverses cytokinesis. Cells do not resume cytokinesis after washing out CalA (upward arrow).

CalA caused acceleration of chromosome movement when added during anaphase (23 cells), at 30 seconds – 8 minutes after anaphase onset, before the chromosomes were half-way to the poles (Table 1; Fig. 3A) [see Additional file 2]. All chromosomes in a cell generally responded the same way (Table 1), all accelerating. In two cells, though, none of the half-bivalents were affected and in one cell they all slowed after CalA, but two of these cells were treated with 5 or 10 nM CalA. In 22/23 cells all pairs of half-bivalents in the cell responded identically; in the one other cell 1 of 3 half-bivalent pairs was not affected by CalA, while the other 2 half-bivalent pairs accelerated.

**Table 1:** Effect of Calyculin A and Okadaic acid on anaphase chromosome movements**

		# of half-bivalent pairs followed				
		
Treatment	# of cells	total	accelerated	slowed	no change	backwards
CalA in anaphase	23	52	45	3	4	29
Okadaic acid in anaphase	6	12	0	3	9	0

** Though there are three half-bivalent pairs per cell, they are often not in the same focal plane; thus the number of pairs for which we have data is less then the maximum number of half-bivalent pairs for a given number of cells.

The sped-up chromosomes moved fast to the poles, with average separation velocities two times higher than average velocities before CalA (Table 2). Some of the chromosomes moved four times faster than before adding CalA. After the half-bivalents arrived at the poles they made short jiggling movements for several minutes. Once at the poles, some chromosomes rapidly moved backwards, toward the equator, without rotating or changing orientation from when they moved poleward. The backwards movements affected the chromosomes randomly. Even when all half-bivalents accelerated poleward, not all of them necessarily moved backwards. Nor did both partners necessarily move backwards: sometimes only one half-bivalent from a pair moved backwards, while the other half-bivalent remained at the pole. Sometimes, when they did move backwards, the half-bivalents from the same pole grouped together and joined the group of half-bivalents coming from the other pole, either in the middle of the cell, or closer to one of the poles, to form what looked like a big nucleus. There was no cytokinesis after CalA treatment, even after the CalA was washed out.

**Table 2:** Velocities after Calyculin A treatment in anaphase (in μm/min)

	Separation velocities			
		
	before calA	after calA	× times faster*	Backward movements
Mean	1.26	2.35	2.07	-2.13
Standard Deviation	0.49	0.78	0.90	0.81
Minimum	0.52	1.05	1.04	-4.11
Maximum	2.63	3.96	4.23	-0.62
Number of pairs	38	38	38	29

* average of individual speeds

The equatorial sex chromosomes also are affected by CalA. In control cells, without exception, sex chromosomes remain stationary at the equator as autosomes move poleward. Immediately after adding CalA during autosomal anaphase, however, the sex chromosomes take long excursions up and down along the spindle, at high speeds and with sudden changes of direction, and they do not segregate in a normal anaphase.

Because of these dramatic effects on the pre-anaphase sex chromosomes, we added CalA to pre-anaphase cells, to see if it would affect autosomes similarly.

### Calyculin A added in metaphase and prometaphase

When CalA is added during *metaphase *(2 cells), 2–4 minutes before anaphase, the chromosomes still entered anaphase, moved with high speeds poleward, and then moved backwards (Fig. 3B). There was no cytokinesis. Thus, the behaviour of the chromosomes in metaphase-treated spermatocytes is the same as in anaphase-treated spermatocytes.

When CalA was added during early to mid-*prometaphase *(13 cells), autosomes moved fast along the length of the spindle, without disjoining, and they jiggled and rotated through up to 180–360° in all three axes, as if they no longer were attached to kinetochore spindle fibres [see Additional file 3]. They did not align at the equator and no metaphase plate formed. These spermatocytes did not enter anaphase (we followed the cells for up to 3 hours). In cells treated with CalA during prometaphase there was no cytokinesis, but otherwise the cells were alive and looked healthy. Sex chromosomes moved up and down along the spindle, similar to their movements in anaphase-treated spermatocytes. In some of the cells, CalA caused blebbing of the plasma membrane.

### Calyculin A added during cytokinesis

Cytokinesis in crane-fly spermatocytes is a myosin-dependent process [1,21], but, as with other cells [76,89], CalA (added 4–7 minutes after the onset of cytokinesis) stopped cleavage almost immediately (it took 2–3 minutes for the effect to be visible) and the furrow regressed (Fig. 3C). Cleavage did not resume after washing out CalA.

### Calyculin A and myosin

CalA might affect PPase1 or PPase2A enzymes as well as myosin phosphatase. We did several experiments to test whether the effects indeed were via myosin phosphorylation. In one set of experiments we treated anaphase cells with 25–50 nM okadaic acid, the same concentration at which we used CalA. Okadaic acid had no effect on chromosome movement or, in a small proportion of pairs (Table 1), slowed or stopped chromosome movement. Thus, since okadaic acid inhibits PPase2A at the same concentration as CalA, but requires 100 times the concentration of CalA to inhibit PPase1 [70], the effect of CalA would seem to be via a PPase1, such as myosin phosphatase.

**Figure 4:**
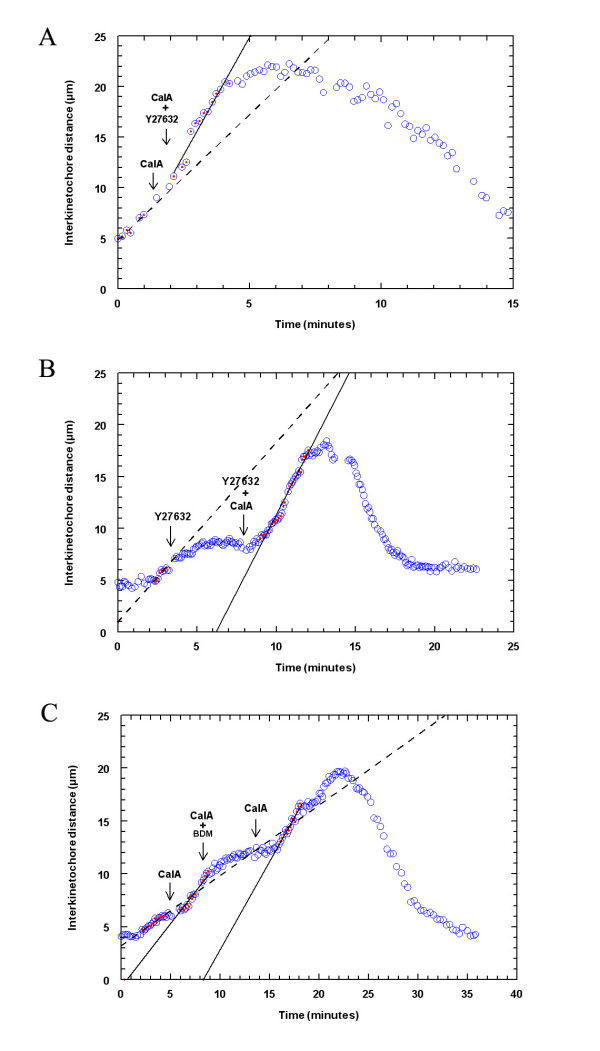
**(A) **Chromosome movement in a cell treated with CalA (left arrow) and with Y-27632 in CalA (right arrow) during anaphase, illustrating one pair of half-bivalents that accelerated after CalA, did not slow after Y-27632, and moved backwards after reaching the poles. The dashed line represents linear regression through the circles with dots before addition of CalA and the solid line through the circles with dots after addition of Y27632 in CalA. **(B) **Chromosome movement in a cell treated with Y-27632 (left arrow) and with CalA in Y27632 (right arrow) illustrating one pair of half-bivalents that stopped after Y-27632, accelerated after CalA and moved backwards after reaching the poles. The dashed line represents linear regression through the circles with dots before addition of Y27632 and the solid line through the circles with dots after addition of CalA in Y27632. **(C) **Chromosome movement in a cell treated with CalA (left arrow), followed by BDM in CalA (mid arrow) and then by CalA again (right arrow) illustrating one pair of half-bivalents that accelerated after Calyculin, slowed after BDM, accelerated again after washing out BDM and moved backwards after reaching the poles. The dashed line represents linear regression through the circles with dots before addition of CalA and the solid lines regression through the circles with dots after addition of CalA (left line) and after washing out BDM (right line).

To further test whether the acceleration and backward movement are due to CalA effects on myosin, we double-treated crane-fly spermatocytes with CalA and one of two myosin inhibitors, Y-27632 (a Rho-kinase inhibitor) or BDM. Rho-kinase [90,91] regulates myosin II activity [1,39] by phosphorylating the myosin regulatory light chain (RLC) and by inhibiting myosin light chain phosphatase, both of which activate myosin. If CalA affects anaphase by preventing MLC dephosphorylation, inhibiting Rho-kinase with Y-27632 should have no effect on chromosome movement if added after CalA, since myosin already will be phosphorylated. Y-27632 slows chromosome movement when added by itself [1], but Y-27632 (in CalA) added to anaphase crane-fly spermatocytes already treated with 50 nM CalA did not slow down the already accelerated anaphase movements (Fig. 4A and Table 3). In the reverse experiment, we added Y-27632 before CalA in anaphase cells. In this experiment the kinase is inhibited first; thus myosin will not be phosphorylated by Rho-K. Were this the only pathway by which myosin could become phosphorylated we would expect that inhibition of phosphatase would not make any difference to the functioning of myosin and that there would be no acceleration. The chromosomes slowed after Y-27632, as expected, but when CalA (in Y-27632) was added, the chromosomes sped up, reaching the same speeds or higher than before addition of Y-27632 (Fig. 4B). The chromosomes also moved backwards (Table 3). Thus, the effects of Y-27632 are overcome by CalA, similar to results in smooth muscle, where addition of CalA after Y-27632 increases force production, effects presumed to be due to phosphorylation of RLC by other enzymes [92].

**Table 3:** Effect of double treatments on anaphase chromosome movements

		# of half-bivalent pairs followed							
		
Treatment	# of cells	total	accelerated after first drug	slowed or stopped after first drug	no change after first drug	accelerated after second drug	slowed or stopped after second drug	no change after second drug	backwards
1^st ^– CalA in anaphase 2^nd ^– Y27632 in anaphase	5	11	9	0	2	0	0	11	5
1^st ^– Y27632 in anaphase 2^nd ^– CalA in anaphase	3	7	0	7	0	7	0	0	2
1^st ^– CalA in anaphase 2^nd ^– BDM in anaphase	3	7	7	0	0	0	6	1	5*
1^st ^– LatB in anaphase 2^nd ^– CalA in anaphase	4	7	0	4	3	7	0	0	3*

* 3 of the 5 half-bivalent pairs are from cells in which the second drug (BDM) was washed out

** 2 of the 3 half-bivalent pairs are from one cell in which LatB had no effect and 1 of the 3 pairs is from a cell in which LatB slowed chromosome movement

To further test whether the effect of CalA on anaphase is due to its action on myosin, we double treated cells with CalA followed by BDM made up in CalA solution. BDM, a myosin inhibitor that acts directly on phosphorylated myosin and inhibits its motor activity [93], stops or slows chromosome movement in crane-fly spermatocytes when added during anaphase [1,21]. When we added 5 mM BDM to CalA treated cells as the chromosomes started to accelerate their poleward movement, the chromosomes slowed down or stopped (Table 3; Fig. 4C). The chromosomes resumed their accelerated movement after washing out BDM but leaving cells immersed in CalA (Fig. 4C).

**Figure 5:**
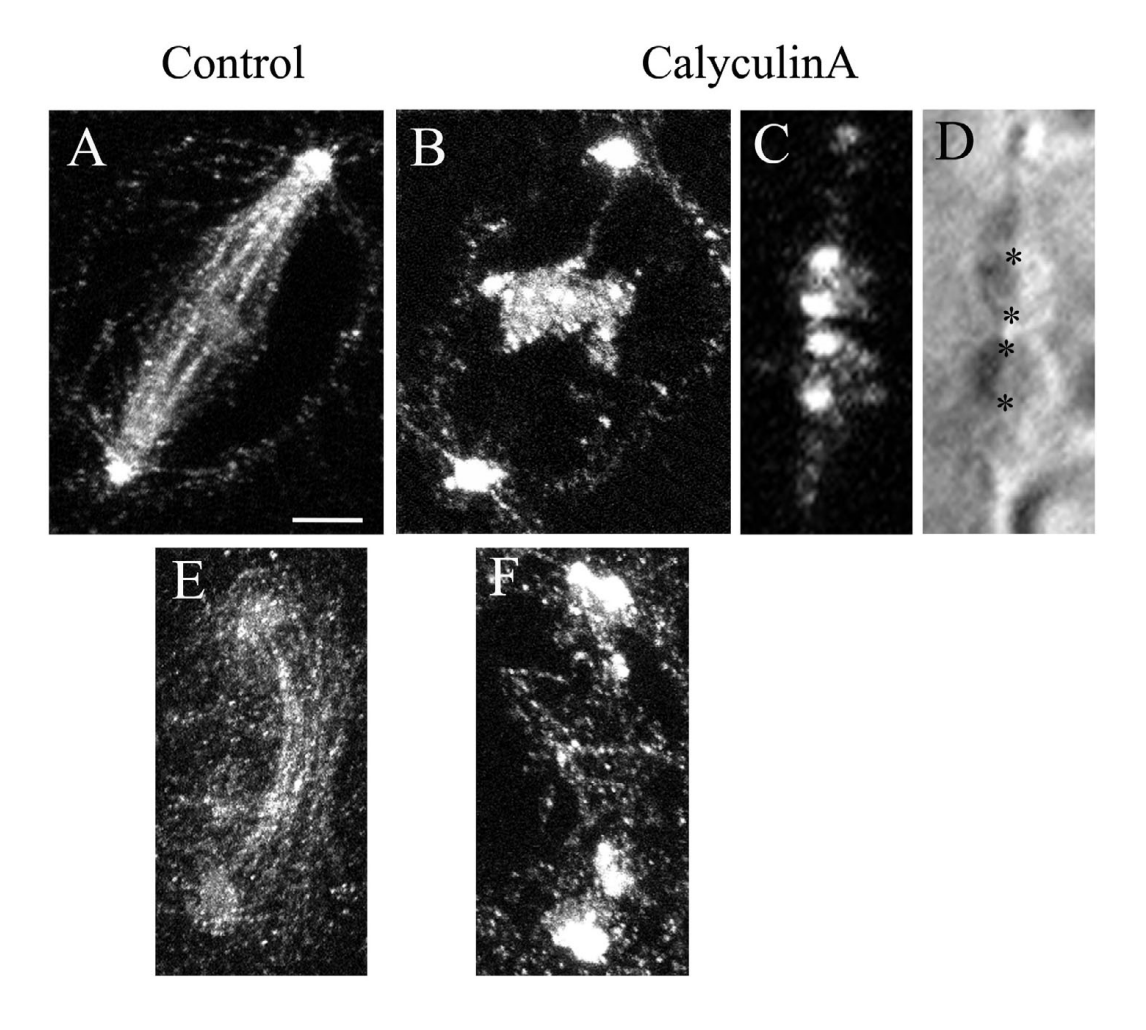
Distribution of phosphorylated myosin RLC (p-squash) in crane-fly spermatocytes. **(A) **Confocal fluorescence micrograph of p-squash in a control crane-fly spermatocyte at metaphase. P-squash localizes to the spindle, at the poles and, with lower intensities, in the chromosomes. **(B) **Confocal fluorescence micrograph of p-squash in a crane-fly spermatocyte treated with 50 nM CalA. P-squash concentrates around the chromosomes and at the poles. From the timing and appearance of the cell, this cell is in the stage after the half-bivalents moved backwards and reformed a pseudo-nucleus. **(C) **Fluorescence micrograph illustrating localisation of p-squash at the kinetochores of the two sex chromosomes, during autosomal anaphase, after CalA treatment. P-squash also localizes at the kinetochores of autosomes. **(D) **DIC image of the cell illustrated in (C) showing the two sex chromosomes and their kinetochores (asterisks). **(E) **Fluorescence micrograph illustrating localisation of p-squash in the midbody and in the daughter nuclei during cytokinesis. **(F) **Fluorescence micrograph illustrating increased staining with antibody against p-squash in the cleavage furrow and in the chromosomes after CalA treatment. Scale bar = 5 μm.

As a further test, we studied the distribution of phosphorylated myosin, using antibodies against the phosphorylated form of squash, the *Drosophila *myosin RLC [94]. In control metaphase or anaphase spermatocytes, phosphorylated squash (p-squash) was restricted to spindle poles and along the spindle fibres (Fig. 5A), and was not present in the cytoplasm, where much of the total myosin is seen [22], suggesting in itself that myosin has a role in chromosome movement. During cytokinesis p-squash staining was seen in the mid body, but was very weak and barely detectable in the cleavage furrow (Fig. 5E). CalA changed the distribution and intensity of staining for p-squash; instead of staining along the chromosomal spindle fibres, staining for p-squash was concentrated around chromosomes and at the poles (Fig. 5B), with spots of high intensity staining at the kinetochores (Fig. 5C, D). Because spindle p-squash was redistributed, it is difficult to be certain if its staining increased after treatment with CalA, but the intensity of cleavage furrow staining was increased compared with the controls (Fig. 5E, F), confirming that treatment with CalA increases phosphorylation of myosin.

### Calyculin A affects the distribution of spindle proteins

Since CalA altered the distribution of p-squash, we used confocal microscopy to study the distribution of various other spindle proteins (i.e. actin, tubulin, titin, skeletor and myosin) in cells treated with 50 nM CalA, to see if CalA caused changes in their distribution as well. Crane-fly spermatocytes were treated with CalA for 3, 5, 10 or 25 minutes, according to the timing of various effects we saw in living cells. In cells treated for 25 minutes the spindle shape was not visible after staining with tubulin and actin. For shorter treatments there was not a drastic difference between 3, 5 and 10 minutes treatment, so we present them together.

**Figure 6:**
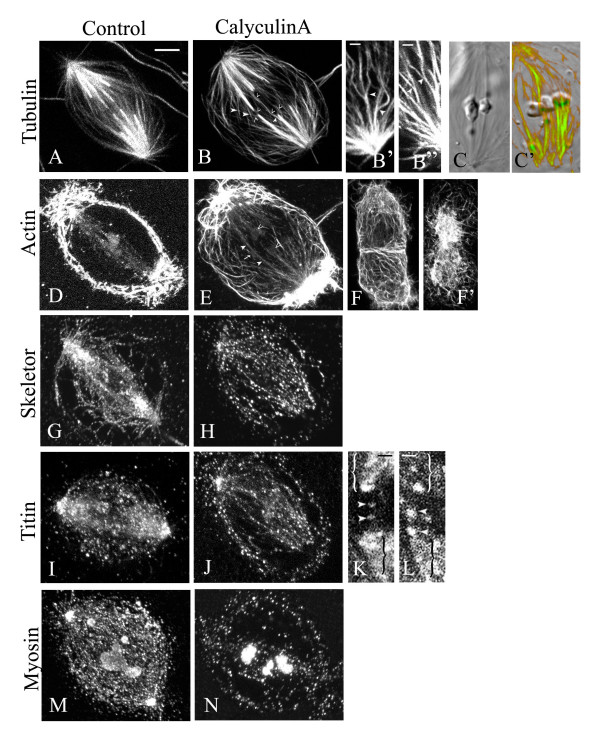
Distribution of various spindle proteins in crane-fly spermatocytes. Control spermatocytes are illustrated in (A,C,D,F,G,I,K,M). Calyculin-treated spermatocytes are illustrated in (B,B',B",C',E,F',H,J,L,N). **(A)**: Fluorescence image of tubulin distribution in a control cell in early anaphase. (**B, B', B"**): Fluorescence images of tubulin distribution in CalA-treated cells in early anaphase (B) and metaphase (B',B"). Individual microtubules "peel off" from the kinetochore bundles (arrowheads), split along their length and surround the chromosomes (open arrowheads) after CalA treatment. **(C)**: DIC image of a control spermatocyte showing kinetochore spindle fibres terminating at the kinetochores. **(C'): **Fluorescence image merged with the DIC image (orange-green) showing that CalA added during prometaphase causes chromosomes to loose attachment to the spindle fibres. **(D): **Fluorescence image of filamentous actin distribution in a control cell in metaphase.**(E): **Fluorescence images of filamentous actin distribution in a CalA-treated cell in early anaphase. Actin filaments become more prominent in the kinetochore fibres. Two half-bivalents from the same autosomal pair (open arrowheads), one univalent sex chromosome (arrow) and sex chromosome spindle fibres (closed arrowheads) are indicated **(F)**: Fluorescence image of a control cell in cytokinesis. **(F')**: Fluorescence image of a cell in cytokinesis after CalA treatment. CalA causes formation of actin aggregates in the mid body. **(G, I, M): **Fluorescence images of skeletor (G), titin (I) and myosin (M) in control cells in prometaphase. **(H, J, N)**: Fluorescence images of skeletor (H), titin (J) and myosin (N) in CalA-treated cells. There is less of these proteins in the spindle region. **(K,L)**: Titin is present in the interzone (arrowheads) between the arms of separating half-bivalents (arrows), both in control cells (K) and in CalA-treated cells (L). Scale bar in A (for panels A-J, M-N) = 5 μm. Scale bar in (B',B",K,L) = 1 μm.

In control cells at metaphase there are prominent, thick bundles of kinetochore fibre microtubules and abundant non-kinetochore microtubules as well (Fig. 6A). In cells treated with CalA for 10 minutes or less, the non-kinetochore microtubules are lost; the kinetochore microtubule bundles are thinner than those in normal cells and are wavy (Fig. 6B,B'B"). Microtubules also seem to leave the kinetochore bundle by detaching at the kinetochore end and "peeling off" at an angle (Fig. 6B,B',B"), which is never seen in control cells. In control prometaphase spermatocytes, the kinetochore microtubule bundles are thinner and much less prominent than at metaphase because of the more abundant non-kinetochore microtubules. In prometaphase cells treated with CalA, kinetochore microtubules are not seen (Fig. 6C'), i.e., the microtubules do not end at the kinetochore, and there seem to be fewer non-kinetochore microtubules than in control cells. Thus it appears that the chromosomes lost their kinetochore attachment to the microtubules, which would explain how they were able to rotate in the spindle. In some of the CalA-treated spermatocytes the spindle fibers lost their focus at the pole and the pole seems to be wider than in control cells (Fig. 6B,C').

*Actin *also is affected by CalA: actin filaments present in the spindle fibres (Fig. 6D) become more "visible" as stronger/thicker bundles of actin filaments (Fig. 6E). We identified continuous actin filaments mainly along the kinetochore spindle fibres (Fig. 6E). Actin filaments in the cortex seem to be shorter and broken, sometimes with a "knotted" appearance. Actin also accumulated at the two poles and there was less actin in the mid region of the cell. The actin filament distribution also was altered in cells in which cytokinesis started before CalA addition: there were numerous actin aggregates in the midregion of the cell or in the midbody (Fig. 6F,F').

*Skeletor*, which normally is arranged in beaded filaments along the spindle fibres (Fig. 6G), also was affected by CalA. In kinetochore microtubules that extended from kinetochore to the pole, the skeletor beads seemed much farther apart than in control cells (Fig. 6H). Skeletor also was associated with the "peeled off" microtubules, but, unlike in non-treated cells, skeletor was not associated with anything other than kinetochore microtubules. Furthermore, there is less skeletor along the interzone connections between half-bivalents and along the sex chromosome kinetochore microtubule bundles than in controls.

*Titin *also is affected by CalA. Titin still is localised primarily in the spindle, but there is less of it than in controls and it seems less well organized (Fig. 6J). Titin remains present in the interzone during anaphase (arrowheads in Fig. 6K, L), distributed between the arms of separating half-bivalent pairs (brackets in Fig 6K, L).

*Myosin*, which normally is present in the whole spindle area and also outside the spindle (Fig. 6M) [22], is affected by CalA in that there is less myosin in the spindle and in the cytoplasm, but more myosin in the chromosomes (Fig. 6N) or around the chromosomes.

### Calyculin A affects myosin in actin-filament-free spindles

**Figure 7:**
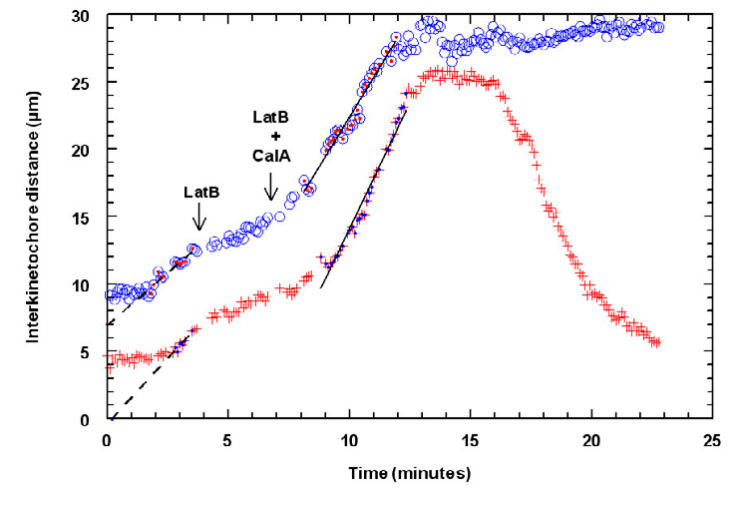
Chromosome movement in a cell treated with LatB (left arrow) and with CalA in LatB (right arrow) illustrating two pairs of half-bivalents that drastically slowed after LatB and accelerated after CalA in LatB. One moved backwards after reaching the poles (+), while the other stayed at the poles (o). The dashed lines represents linear regression through the dotted circles and crosses before addition of LatB and the solid lines regression through the dotted circles and crosses after addition of CalA in LatB.

We have shown previously that spindles *in vivo *can function normally even when filamentous actin is removed in prometaphase [1]. Because anti-myosin drugs still altered chromosome movements in these spindles, we argued that myosin might work against kinetochore microtubules. Thus it was of interest to test whether CalA still could cause acceleration even in the absence of actin filaments. First, we studied the effects of LatB and CalA added together in anaphase. When LatB was added in early anaphase, 1.3–4.3 minutes after anaphase onset, chromosomes slow their motion (Table 3), but after CalA was added in mid-anaphase, in the continuing presence of LatB, at about 3 minutes after LatB, anaphase movements sped up (Fig. 7) and backwards movement sometimes occurred (Table 3). Thus the CalA effects do not seem to require the presence of filamentous actin.

We then added CalA to actin-filament-free spindles, formed by adding LatB to cells in early prometaphase. CalA (in LatB) added to actin-filament free spindles in early anaphase, 2–7 minutes after anaphase onset, gave variable results (Table 4): sometimes chromosomes sped up poleward and then moved backwards, while other times the chromosomes moved backwards immediately, without speeding up to the poles first, the latter occurring primarily in cells in which anaphase movements were slower.

**Table 4:** Effect of Calyculin A on anaphase chromosome movements in actin filament-free spindles

		# of half-bivalent pairs followed						
		
		Initial anaphase movement			Effect of CalA			
		
Treatment	# of cells	total	normal anaphase	slower* anaphase	accelerated after CalA	slowed or stopped after CalA	immediately backwards	backwards
1^st ^– LatB in prometaphase 2^nd ^– CalA in anaphase	5	11	6	5	4	1	6	3

* Slower anaphase refers to separation velocities of less than 0.3 μm/min [101,110,112]

### Backwards movements

**Figure 8:**
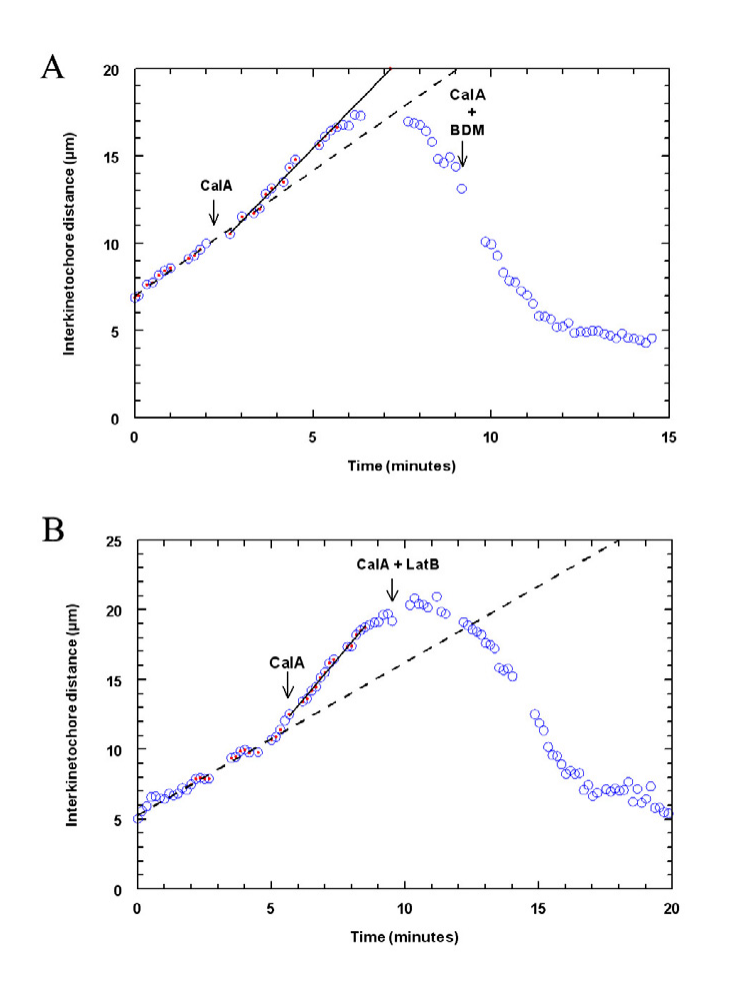
Backwards chromosome movements in crane-fly spermatocytes treated with CalA are not actin or myosin dependent. **(A) **Chromosome movements in a cell treated with CalA in anaphase (left arrow), and with BDM in CalA added after the chromosomes started to move backwards (right arrow). Backwards movements are not affected by the myosin inhibitor BDM. The dashed line represents linear regression through the circles with dots before addition of CalA and the solid line through the circles with dots after addition of CalA. **(B) **Chromosome movements in a cell treated with CalA in anaphase (left arrow), and with LatB in CalA added after the chromosomes reached the poles, at the time when they started to move backwards (right arrow). Backwards movements are not affected by actin inhibitor LatB. The dashed line represents linear regression through the circles with dots before addition of CalA and the solid line through the circles with dots after addition of CalA.

An unexpected result of CalA added in anaphase or metaphase crane-fly spermatocytes was the backwards movement of the half-bivalents after they reached the poles or, in actin-filament free spindles, immediately after CalA addition. Previous studies have suggested that there are elastic tethers that connect the separating half-bivalents and that these tethers produce backwards forces which act on autosomal half-bivalents at anaphase [95]. We tested whether the backwards movements caused by CalA are due to myosin and actin. We treated cells with CalA during early anaphase, 1.3 – 5.3 minutes after anaphase onset; when the chromosomes started to move backwards we added either BDM at 5 mM (a concentration at which BDM blocks anaphase) [1] or at 20 mM, or we treated them with 1.5 μM LatB. Neither of these inhibitors had any effect on backwards movement of chromosomes (Table 5; Fig. 8A, B). These results suggest that backward movements are not driven by actin or myosin, but could be rather a mechanical process, like elastic tethers 'pulling" back the separated chromosomes, as suggested by LaFountain et al. [95]. It also is conceivable that the chromosomes slide backwards along interzonal microtubules, but this conclusion is not supported by our observations in living cells. The half-bivalents do not rotate after they reach the poles; they move backwards with their arms leading, not with their kinetochores, as one would expect if the backward movement were due to chromosomes sliding along microtubules. Thus it is unlikely that the backwards movements are due to microtubules. Titin, on the other hand, could be part of the "tethers" pulling the chromosomes back since the distribution of titin molecules between the arms of the half-bivalent pairs is not altered after CalA treatment (Fig. 6K, L).

**Table 5:** Effect of actin and myosin inhibitors on backwards movement in CalA-treated spermatocytes

		# of half-bivalent pairs followed			
		
Treatment	# of cells	total	backwards	Slowed or stopped after BDM or LatB	no change after BDM or LatB
1^st ^– CalA in anaphase 2^nd ^– BDM or LatB in late anaphase	7	15	12	0	12

## Discussion

In this study we showed that CalA, an enhancer of myosin, added during anaphase or metaphase, speeds up anaphase chromosome movement. Subsequently chromosomes move backwards. CalA also causes sex chromosomes to make long excursions in the spindle at high speed, and to rotate; and it blocks cytokinesis. When added during prometaphase, chromosomes rotate, move up and down, and jiggle. CalA also affects the distribution of various spindle proteins.

A major finding of our study is that anaphase chromosome movements were accelerated by a factor of two after addition of CalA. We think it likely that this acceleration is due to hyper-phosphorylation of myosin. An alternate possibility is that the acceleration is due to CalA action on other possible targets. Okadaic acid blocks PPase2A activity at the same concentration as CalA [70], and since okadaic acid did not cause either acceleration or backwards movements, this would seem to eliminate the possibility that PPase2A is a CalA target. It is possible that CalA acts on a PPase1 other than on the myosin phosphatase, but several lines of evidence speak against this. For one, the general effect of inhibiting random PPases is that cells do not proceed past metaphase [61], or are much delayed [97], whereas crane-fly spermatocytes treated in metaphase with CalA entered anaphase normally and chromosome movement was accelerated, arguing that the CalA effect is a more specific one. For another, staining with antibodies to p-squash indicates that phosphorylated myosin is located in the spindle, along the kinetochore microtubule bundles; staining with antibodies against various PPase1 isoforms indicates that one such enzyme also is located along spindle fibres [60]. CalA causes hyper-phosphorylation of myosin, as indicated by increased staining of cleavage furrow myosin and of chromosomes. While none of these arguments is airtight, we nonetheless favour the interpretation that acceleration is due to increased myosin activity because it is consistent with other experiments in which myosin and actin inhibitors block chromosome movement [1,15]. Whereas the interpretation of each individual study or of the action of each individual drug might be debated, the overall fact that inhibitors of actin and myosin generally inhibit movement and a myosin enhancer speeds up movement makes a strong case for involvement of myosin in anaphase chromosome movements and supports our interpretation that the acceleration is caused by myosin hyper-activation. Thus, while we cannot definitively rule out interpretations in which other PPase1 enzymes are involved, we think it likely that chromosome acceleration is due to enhanced myosin activity.

Our interpretation that CalA effects are due to blocking MLCPase would seem to be negated by our experiments using Y27632. Y27632, a specific inhibitor of Rho-K, slowed chromosome movement in anaphase. This effect presumably is due to reduced myosin phosphorylation, and therefore myosin is less active after Y27632 addition. CalA was not expected to accelerate these chromosomes, since myosin was not phosphorylated, but it did. The interpretation of these results is ambiguous, however, because the same result was obtained in studying smooth muscle contraction, which is known to be due to myosin activity [92]. It seems that inhibition of MLCPase by CalA unmasks another phosphorylation pathway, separate from Rho-K pathway and therefore not inhibited by Y-27632 [68]. This is confirmed in other experiments in which inhibition of smooth muscle Rho-K by Y27632 or H-1152 unmasked an integrin-linked kinase which then phosphorylated myosin [55]. Thus, because of such potential redundant phosphorylation pathways, this particular experiment is ambiguous and is not a clear test of how CalA causes chromosome acceleration.

The only previous data on CalA effects on mitosis that we know of is by Hamaguchi and Kuriyama [89]. The authors concluded that anaphase chromosome movements in sand dollar eggs were blocked by okadaic acid and CalA. With respect to CalA, they state in the text that chromosome movements were inhibited by CalA at concentrations ≥ 1–2 μM. We generally used concentrations 20 times lower than that, but we found that concentrations of 0.5 μM caused chromosomes to accelerate. Thus there appears to be a discrepancy between the two sets of results. However, from the description in Figure 10 of [89], it would seem that the authors derived their conclusion from fluorescence micrographs of chromosomes positions after injection of CalA. Their description in Figure 10 that "chromosome movement did not occur, although the chromosomes aggregated into two clusters", does not necessarily have to be due to blocked movements, though; the clusters could have arisen from backwards movements of the kind we described. With respect to the apparent discrepancy between our "no effect" of okadaic acid (Table 1) and the blockage of movement by okadaic acid reported by Hamaguchi and Kuriyama [89], their blockage of movement occurred at concentrations of 1–2 μM, which is 20–40 times higher than the concentration we used (50 nM). Indeed, they found that 50 nM okadaic acid had little or no effect on anaphase in their cells, so there is no discrepancy between the two sets of results.

CalA alters the distribution of various spindle proteins in crane-fly spermatocytes. P-squash staining relocates in the cell, from being associated with the kinetochore microtubules to being at the poles, at the kinetochores, and around the chromosomes. Similar results occur in other systems. For example, there are increased levels of phosphorylated myosin after CalA treatment in various cells [68,69,72,74,75,84] and CalA causes relocation of actin, myosin and other cytoskeletal components [83,98,99]. In crane-fly spermatocytes the microtubule bundles become thinner, presumably due to splitting, narrowing and disappearance of microtubules from the bundles. This is consistent with results in other studies that showed that microtubules are disassembled after CalA [98,100]. In crane-fly spermatocytes actin filaments become more visible in spindle fibres possibly due to actin filament stabilization by inhibition of MLCPase, similar to results in sea urchin eggs [69,76].

When CalA is added in prometaphase, chromosomes in crane-fly spermatocytes lose their attachment to microtubules (Fig. 6C). In our experiments, the unattached chromosomes rotated and moved rapidly up and down in the spindle, similar to the rapid movements displayed by the sex chromosomes after CalA treatment during anaphase. The longitudinal movements might be due to chromosomes capturing and sliding along remnant microtubules, but we can only speculate on forces that might be producing the rotations. All these extraordinary movements, though, seem to indicate that there is loss of equilibrium and lack of coordination between different force producers.

CalA caused late anaphase chromosomes to move backwards. Backward chromosome movements in anaphase have been seen previously in crane-fly spermatocytes, but only rarely and only when the poleward force was blocked by UV microbeam irradiation [101,102]. However, chromosome arms, severed with a laser beam, regularly moved backwards [95]. Backward movements were seen also in silkworm spermatocytes after UV irradiation of a spindle pole [103] : the chromosomes associated with the irradiated pole moved across the equator to the opposite pole. In grasshopper spermatocytes [104] there are fast backwards movements after UV irradiation of the kinetochore in early anaphase. Ilagan and Forer [101], LaFountain et al. [95] and Wong and Forer [102] all considered that there are mechanical connections between the arms of separating half-bivalents in crane-fly spermatocytes and that these connections, or "tethers"[95], elastically cause the backwards movements. Even though the tethers act on both partner half-bivalents, the backwards movements need not be symmetric, with both partners moving equally, because in all previous observations only one chromosome (or arm) moved, the one no longer attached to the pole. We think the same applies to CalA treated cells: when the connection to the pole is lost, then that chromosome moves to the equator independent of the partner at the other pole. We assume that the accelerated movements to the pole are associated (for some chromosomes) with pre-mature release of attachment to the pole, which allows the backward movements to be driven by the elastic tethers between partners. In our experiments, backwards movements in CalA treated spermatocytes were not altered by LatB or BDM, suggesting that the movements are not dependent on actin or myosin. It is possible that actin filaments remaining in the interzone after CalA treatment (e.g., Fig. 6E) are resistant to LatB by virtue of the bundling induced by Cal A, and that the backwards movements might require these actin filaments. Two experiments speak against this possibility, however. For one, we know that LatB removes actin filaments from normal cells [1] yet backwards movements still take place when cells pre-treated with LatB are treated with CalA (Table 3, 4). This indicates that actin filaments are not necessary for backwards movements to take place. For another, BDM has no effect on backwards movements, indicating that those movements do not require myosin or actomyosin. Thus our results indicate that backwards movements require neither actin nor myosin. On the other hand, titin, the protein responsible for muscle elasticity [105-107] is present between the arms of separating half-bivalents in control crane-fly spermatocytes as well as in CalA treated spermatocytes and could provide the necessary elasticity. Thus, our interpretation is that backwards movements are due to titin filaments which pull the chromosomes back together. As a corollary, we suggest that the regularly organized spindle matrix and cytoskeletal components prevent the backwards movement in non-treated cells, but the disorganization that ensues after CalA treatment reduces or removes attachment to the pole and allows titin to pull the chromosomes back together, to near the equator.

## Conclusion

In this study we showed that CalA, an enhancer of myosin, has multiple effects on chromosome movements during anaphase and alters the distribution of spindle proteins in crane-fly spermatocytes. CalA speeds up anaphase chromosome movement when added during anaphase or metaphase. It blocks the formation of the new nuclei in the daughter cells, due to the chromosomes moving backwards, toward the equator, after they reached the poles. When added during prometaphase, chromosomes rotate, move up and down, and jiggle. Sex chromosomes are also affected by CalA, in such that they make long excursions in the spindle at high speed, and rotate. And finally, CalA blocks cytokinesis initiation and completion in crane-fly spermatocytes. We suggest that these CalA effects are via myosin, as indicated by several lines of evidence we discussed.

## Materials and methods

Living crane-fly spermatocytes (*Nephrotoma suturalis *Loew), held in place in a fibrin clot prepared as described in [108], were observed using phase-contrast microscopy with an objective of NA = 1.3, and images were recorded on DVDs in real time. Cells were perfused with insect Ringer's solution or with Calyculin A (LC Laboratories, MA) in insect Ringer's solution, at final concentrations of 0.5 μM, 100 nM, 50 nM, 20 nM, 10 nM or 5 nM, prepared from a 1 mM or a 50 μM Calyculin A stock in DMSO or with okadaic acid (Calbiochem) at final concentrations of 20 nM or 50 nM prepared from a 50 μM stock in DMSO. In addition to CalA and okadaic acid we perfused cells with 50 μM Y27632 (Calbiochem), 5 mM or 20 mM BDM (Sigma), and 1.5 μM LatrunculinB (Calbiochem), each one diluted in a solution containing 50 nM Calyculin A. BDM and Y27632 stocks were made in insect Ringer's solution and LatB stock was made in DMSO. In no experiment was the DMSO concentration greater than 0.2%, which has been shown previously to have no effect on chromosome velocities [14,96,109,110].

For immunostaining we followed the protocol described in detail in [1] using the following solutions: 2.2 μM Alexa 488 phalloidin (Molecular Probes) for filamentous actin; 1:4000 YL1/2 rat antibody against tyrosinated tubulin [111], followed by 1:200 Alexa 594 goat anti rat (GAR); 1:200 My21 mouse IgM antibody against myosin RLC (Sigma), followed by 1:200 Alexa 488 GA mouse IgM; 1:100 1A1 mouse IgM antibody against skeletor [26], followed by 1:200 Alexa 488 GA mouse IgM; 1:500 LP06352 rabbit antibody, followed by 1:200 Alexa 568 GA rabbit; 1:500 α-KZ rat antibody against D-titin, followed by 1:200 Alexa 594 GAR (primary antibodies against titin are a gift from Dr. Debbie Andrew, Johns Hopkins University, MD) [23] ; and 1:400 rabbit anti-phosphorylated myosin squash [94], followed by 1:200 Alexa 568 GA rabbit. All secondary antibodies are from Molecular Probes. For preparations using triple-channel staining, the three secondary antibodies had the following excitation/emission peaks: Alexa 488/519, Alexa 568/602 and Alexa 633/647. Cells were examined with an Olympus Fluoview 300 confocal microscope, the images were collected with Fluoview (Olympus) software, and were processed further using Image J [112]. Illustrations presented in this paper were obtained using Adobe Photoshop and were adjusted for brightness and contrast only.

Chromosome movements in living cells were analysed from images time-lapsed from the DVDs using Virtual Dub [113] as described in [114]. Graphs were plotted using SlideWrite.

## Competing interests

The author(s) declare that they have no competing interests.

## Authors' contributions

JT made the initial observations on the effects of Calyculin A on several anaphase cells while working as a summer NSERC student. LF did the rest of the experimental work, did the bulk of the writing and the preparation of the final manuscript. AF conceived the study, treated some cells with calyculin, participated in the design of the study and analysis of the data, and edited the manuscript.

## Supplementary Material

Additional file 1 Movie representing meiosis I in control crane-fly spermatocyte. Two half-bivalent pairs (out of the total three) are in focus. After the autosomes reached the poles, the two smaller univalents sex chromosomes, which stayed at the metaphase plate, start their anaphase, at about the same time as cytokinesis starts. This movie displays time-lapsed images, obtained from real-time sequences recorded on DVD, played back at 120× the recorded speed. Click here for file

Additional file 2 Anaphase in a crane-fly spermatocyte treated with 50 nM Calyculin A at 13:03:30. Two half-bivalent pairs are in focus. Autosomes accelerated their poleward movement after Calyculin A addition. After they reached the poles, they moved backwards, toward the equator, where they joined their partners from the opposite pole and the two univalent sex chromosomes. Cell does not enter cytokinesis. This movie displays time-lapsed images, obtained from real-time sequences recorded on DVD, played back at 120× the recorded speed. Click here for file

Additional file 3 Prometaphase in a crane-fly spermatocyte treated with 50 nM Calyculin A at 13:41:00. Two autosomal bivalents are in focus. After Calyculin A addition, the chromosomes started to rotate and to move randomly in the spindle, much faster than before Calyculin A. The chromosomes do not align at the metaphase plate and the cell does not enter anaphase. This movie displays time-lapsed images, obtained from real-time sequences recorded on DVD, played back at 120× the recorded speed. Click here for file
